# Low dose aspirin prevents endothelial dysfunction in the aorta and foetal loss in pregnant mice infected with influenza A virus

**DOI:** 10.3389/fimmu.2024.1378610

**Published:** 2024-04-04

**Authors:** Madison Coward-Smith, Stella Liong, Osezua Oseghale, Jonathan R. Erlich, Mark A. Miles, Felicia Liong, Kurt Brassington, Steven Bozinovski, Ross Vlahos, Robert D. Brooks, Doug A. Brooks, John J. O’Leary, Stavros Selemidis

**Affiliations:** ^1^ Centre for Respiratory Science and Health, School of Health & Biomedical Sciences, Royal Melbourne Institute of Techology (RMIT) University, Melbourne, VIC, Australia; ^2^ Clinical and Health Sciences, University of South Australia, Adelaide, SA, Australia; ^3^ Discipline of Histopathology, School of Medicine, Trinity College Dublin, Dublin, Ireland; ^4^ Sir Patrick Dun’s Research Laboratory and the Trinity Translational Medicine Institute (TTMI), St. James’s Hospital, Dublin, Ireland

**Keywords:** pregnancy, influenza, vascular dysfunction, aspirin, foetal

## Abstract

Influenza A virus (IAV) infection in pregnancy resembles a preeclamptic phenotype characterised by vascular dysfunction and foetal growth retardation. Given that low dose aspirin (ASA) is safe in pregnancy and is used to prevent preeclampsia, we investigated whether ASA or NO-conjugated aspirin, NCX4016, resolve vascular inflammation and function to improve offspring outcomes following IAV infection in pregnant mice. Pregnant mice were intranasally infected with a mouse adapted IAV strain (Hkx31; 10^4^ plaque forming units) and received daily treatments with either 200µg/kg ASA or NCX4016 via oral gavage. Mice were then culled and the maternal lungs and aortas collected for qPCR analysis, and wire myography was performed on aortic rings to assess endothelial and vascular smooth muscle functionality. Pup and placentas were weighed and pup growth rates and survival assessed. IAV infected mice had an impaired endothelial dependent relaxation response to ACh in the aorta, which was prevented by ASA and NCX4016 treatment. ASA and NCX4016 treatment prevented IAV dissemination and inflammation of the aorta as well as improving the pup placental ratios *in utero*, survival and growth rates at post-natal day 5. Low dose ASA is safe to use during pregnancy for preeclampsia and this study demonstrates that ASA may prove a promising treatment for averting the significant vascular complications associated with influenza infection during pregnancy.

## Introduction

Influenza A infection (IAV) during pregnancy is a serious health concern for both the mother and developing neonate. IAV infection has been linked to a significantly higher risk of hospitalisation, preterm birth and low birth weights as well as miscarriages, stillbirths and early neonatal disease and death (1, 2). Long term neurological effects on the baby have also been associated with maternal influenza infection such as the development of schizophrenia, autism spectrum disorder and Parkinson’s disease (3, 4). IAV infection differs from other viruses such as Zika or Rubella (5, 6), as vertical transmission across the placenta to the foetus is rare (7), and therefore the effects on the developing foetus are not attributed to the virus directly. Despite these health complications for both mother and offspring, the mechanisms underpinning IAV pathology during pregnancy remain largely unknown and consequently there are limited treatment options once an infection has established. Recently, we have shown that IAV infection causes maternal immune activation that disrupts normal maternal vascular function with a particular dysfunction of the endothelium of the aorta (8). These findings highlight that IAV infection during pregnancy presents a significant cardiovascular phenotype, which could be targeted by a myriad of possible therapeutic options that have well established beneficial properties for the cardiovascular system.

During pregnancy, significant alterations occur to the maternal cardiovascular system to support the growing foetus. This typically begins at 8 weeks and causes an increase in the total cardiac output by 20%, resulting from increased peripheral vasodilation (9). By 24 weeks, this is further increased to 45% in a healthy pregnancy (10). Abnormal maternal cardiovascular function during pregnancy can result in severe health complications and even death to both the mother and the developing foetus. Approximately 10% of all pregnancies are affected by hypertensive disorders (11), which can affect the cardiovascular health of the mother. One of the most well studied hypertensive disorders in pregnancy is preeclampsia, which is defined as the onset of hypertension past 20 weeks of gestation and results in systemic peripheral vascular inflammation and oxidative stress. This can lead to impaired maternal blood flow and the development of placental insufficiency. Placental insufficiency is defined as an inability of the placenta to provide enough nutrients and oxygen to the developing foetus (12) and is associated with foetal complications including intrauterine growth restriction (IUGR), foetal hypoxia, preterm birth, foetal distress and death (13). Long term effects of placental insufficiency on the foetus have even been linked with an increase in risk of mortality from ischaemic heart disease and increased risk of stroke in adult life (14). We have previously established that IAV-infected pregnant mice exhibited a severe ‘vascular storm’ phenotype characterised by vascular inflammation. In particular IAV causes an inflammatory condition that manifests within the perivascular adipose tissue compartment and results in endothelial dysfunction of the aorta (8); coinciding with the detection of significant amounts of IAV within the aorta. We also observed that offspring and placentas from infected dams were significantly smaller compared to uninfected dams, and expressed higher levels of hypoxic and angiogenic markers, suggesting that the vascular storm affected not only the mother’s cardiovascular health, but also impacted the placenta and developing foetus. Current treatments for IAV infection during pregnancy include vaccination for prevention of infection or the administration of anti-virals, such as Oseltamivir, however both strategies have their limitations. Despite vaccination being strongly recommended to pregnant women, vaccination rates remain low ranging from 40-60% (15–17) while issues with anti-viral medications include short administration windows and anti-viral resistance (18). The cardiovascular phenotype observed during maternal influenza infection lends itself to a pharmacological strategy of using safe drugs that are well regarded to influence the cardiovascular system, which may help to improve vascular function and improve offspring outcomes.

Aspirin or acetylsalicylic acid (ASA) is commonly prescribed as a non-steroidal anti-inflammatory medication and is commonly prescribed at low doses (between 50 and 150mg/day (19)) for pregnant women who are at-risk or have developed preeclampsia (20). Studies show that women who are prescribed low dose ASA (LDA) before 16 weeks, show effective secondary prevention of preeclampsia (21). ASA supresses the production of prostaglandins and thromboxanes through its ability to irreversibly inactivate cyclooxygenases (22). By doing this, it prevents inflammation. ASA is also prescribed commonly as a long-term treatment option for individuals with cardiovascular disease (23) due to its anti-thrombotic properties and ability to induce endothelial-dependent relaxation of blood vessels, by increasing nitric oxide release (24). The effect of ASA and its ability to combat IAV-induced inflammation and viral replication is poorly reported, with some studies suggesting ASA is effective *in vitro* in reducing viral titres (25), while there is a general lack of ASA studies *in vivo* using viral murine models. NCX4016, a novel nitric oxide releasing derivative of ASA, is also a potent anti-thrombotic agent. Synthesised primarily to overcome gastrointestinal issues associated with ASA prescription (26), at low doses, NCX4016 (30mg/kg) has been shown to be as effective as high dose ASA (54mg/kg) in reducing hypercholesteremia in mice (27). However, there are currently no studies involving NCX4016 in pregnancy and its efficacy during influenza infection.

Thus, the aim of the present study was to interrogate whether ASA or NCX4016 treatments could reverse the vascular dysfunction caused by IAV infection in pregnant mice and whether this improves offspring outcomes.

## Materials and methods

### Ethics

All animal experimentation was approved by the Animal Ethics Committee (AEC) at RMIT University (Animal Ethics Committee Number 1801) and as specified by the guidelines of the Australian Code of Practice for the Care of Experimental Animals and National Health and Medical Research Council of Australia (NHMRC).

### Virus

Mouse-adapted HKx31 IAV strain (X31; H3N2) was obtained from Professor Patrick Reading, Department of Immunology and Microbiology, The Peter Doherty Institute for Infection and Immunity, University of Melbourne. Viral aliquots were stored at -80°C until the day of use, where they were thawed and diluted in phosphate buffered saline (PBS) as a working stock for infection.

### Aspirin and NCX preparation

Commercially available aspirin (ASA) tablets (Aspro Clear Regular Strength; Bayer Australia) was used in this study. The list of excipients present in the Aspro Clear Regular Strength included: citric acid, docusate sodium, Flavour, malic acid, mannitol, povidone, saccharin sodium, sodium bicarbonate and sodium carbonate. ASA tablets were dissolved in 1% dimethyl sulfoxide (DMSO) in PBS for a final concentration of 200 μg/mL. Aliquots were made and stored at -80 °C until use. NCX4016 (Sigma-Aldrich) was dissolved in DMSO. Following this, NCX4016 was diluted in PBS to gain a stock concentration of 200 μg/mL in 1% DMSO. Aliquots were then frozen at -80 °C until use. Vehicle control solution was prepared using 1% DMSO in PBS.

### Infection and treatment

Time-mated pregnant C57BL/6 mice (total number was 108) were purchased from Animal Research Centre (Perth, Western Australia) and then randomly assigned to equal size experimental groups. At embryonic day (E)12 gestation mice were sedated with isoflurane and intranasally infected with 35 μL of 10^4^ plaque forming units (PFU) of the HKx31 IAV strain (X31) as previously described (8). Some mice were not infected with X31 but were given PBS. Mice were weighed daily and administered with either 200 μg/kg bodyweight of (i) ASA, (ii) NCX4016 or (iii) vehicle control (1% DMSO in PBS). Treatments were given daily via oral gavage from the day of infection, until either 2 or 5 days post infection (dpi), as indicated. Following infection, mice were humanely culled at either 3 or 6 dpi, or 5 days post-partum with ketamine/xylazine mixture (180 mg/kg and 32 mg/kg, respectively) via intraperitoneal injection. Individual pup weights and crown-to-rump lengths were measured in postnatal day (P)5.

### BALF collection and differential cell analysis

Bronchoalveolar lavage fluid (BALF) was extracted to determine levels of inflammation within the airways. Cold PBS was flushed into the lungs of mice through a small incision in the trachea; briefly, a 19-gauge needle was inserted into the incision and 4 volumes of PBS (1x400 µL and 3x300 µL) flushed into the lungs. The number of live cells in the BALF collected was counted using a haemocytometer.

Fifty thousand cells were then centrifuged at 400 x*g* for 5 minutes onto a frosted slide using a Cytospin 3 (Shandon) for differential cell analysis. Slides were fixed with 2-propan-2-ol for 1 minute and left to dry overnight. Slides were then submerged in rapid 1 dye for 12 dips and then immediately placed into rapid II for 6 dips (Shandon Kwik Diff; Thermofisher Scientific). Slides were then left to dry before applying a small drop of DPX mounting media to the cytospot and applying a coverslip. Slides were then imaged under a microscope with 20x magnification. A total of 500 cells were counted from random fields, including macrophages, lymphocytes and neutrophils, based on standard morphological criteria.

### Gene expression analysis

Maternal lungs and aorta were harvested and snapped frozen in liquid nitrogen. RNA was extracted from the tissues using the RNeasy Mini Kit as per manufacturer’s instructions (Qiagen). RNA concentration was then determined using the Nanodrop One (Thermofisher Scientific). Between 1 µg – 2 µg of RNA was then converted to complementary DNA (cDNA) using the Applied Biosystems High-Capacity cDNA reverse transcription kit (Thermofisher Scientific). RNA was then added to the cDNA reverse transcription mastermix to a final volume of 20 µL, and then transcribed using the following settings: 25 °C for 10 minutes, 37 °C for 120 minutes and 85 °C for 5 minutes in a VeritiPro Thermal cycler (Thermofisher Scientific). Samples were then stored at -20 °C until use.

Real-time quantitative PCR (qPCR) was then performed using the Taqman Gene Expression Master mix (Thermofisher Scientific) or SYBR Green PCR Master Mix (Thermofisher Scientific). Analysis was performed using the Quantstudio 7 (Thermofisher Scientific). Pre-designed Taqman primers for RPS18, IL-1β, TNF-α and IL-6 were purchased from Applied Biosystems. Viral titres were measured by SYBR Green using an oligonucleotide sequence for the forward and reverse primers of the segment 3 polymerase (PA) of influenza virus as previously described (28). All quantitative values were obtained using the threshold cycle (Ct) value and then analysed using the comparative Ct method. Fold changes were calculated based on either the control (PBS) or X31 group, which was set to 1.

### Myography

Maternal aortic rings were placed in ice cold physiological Krebs solution (119 mM NaCl, 1.17 mM MgSO_4_, 25 mM NaHCO_3_, 1.18 mM KH_2_PO_4_, 5.5 mM C_6_H_12_O_6_ and 2.5 mM CaCl_2_). Perivascular adipose tissue surrounding the vessel was trimmed off by fine dissection and a 2 mm section of the vessel was taken for analysis and placed in carbogen bubbled Krebs solution. Maximum contractions were obtained using 9,11-dideoxy-9α,11α-methanoepoxy-prosta-5Z,13E-dien-1-oic acid (U46619; Cayman Chemical Company). Concentration response curves to acetylcholine (Ach) to measure vascular endothelium-dependant contractions or sodium nitroprusside (SNP) to measure vascular endothelium-independent relaxation were obtained in aorta pre-contracted to 50% of their respective maximum contractions to U46619. LabChart 7 (AD Instruments) was used for analysis.

### Soluble FLT-1 ELISA

Blood from dams was collected into vacutainer EDTA tubes via cardiac puncture using a 26 G needle and 1ml syringe. Plasma was flash frozen in liquid nitrogen and stored at -80°C until needed. Circulating levels of FLT-1 in plasma samples were quantified with a commercially available sFLT-1 ELISA kit as per manufacturer’s instructions (cat# DY471, R&D Systems).

### Statistical analysis

Data are represented as the mean +/- the standard error of the mean (SEM). All statistical analysis was compared to HKx31 control mice, except when otherwise stated. Statistical analysis was performed using Graphpad Prism software (Version 8). Where appropriate, data found to be not normally distributed was log-transformed to generate normally distributed data for parametric testing. Unless specified otherwise, one-way ANOVA using Dunnett’s multiple comparison post-hoc test was used. Bodyweight and myograph analysis were performed using two-way repeated measures ANOVA with Dunnett’s or Tukey’s multiple comparison post-hoc test. Statistical significance was defined as *P*<0.05.

## Results

### X31 infection significantly hinders pregnancy weight gain, while treatment with ASA and NCX did not affect dam weight

**Figure 1:**
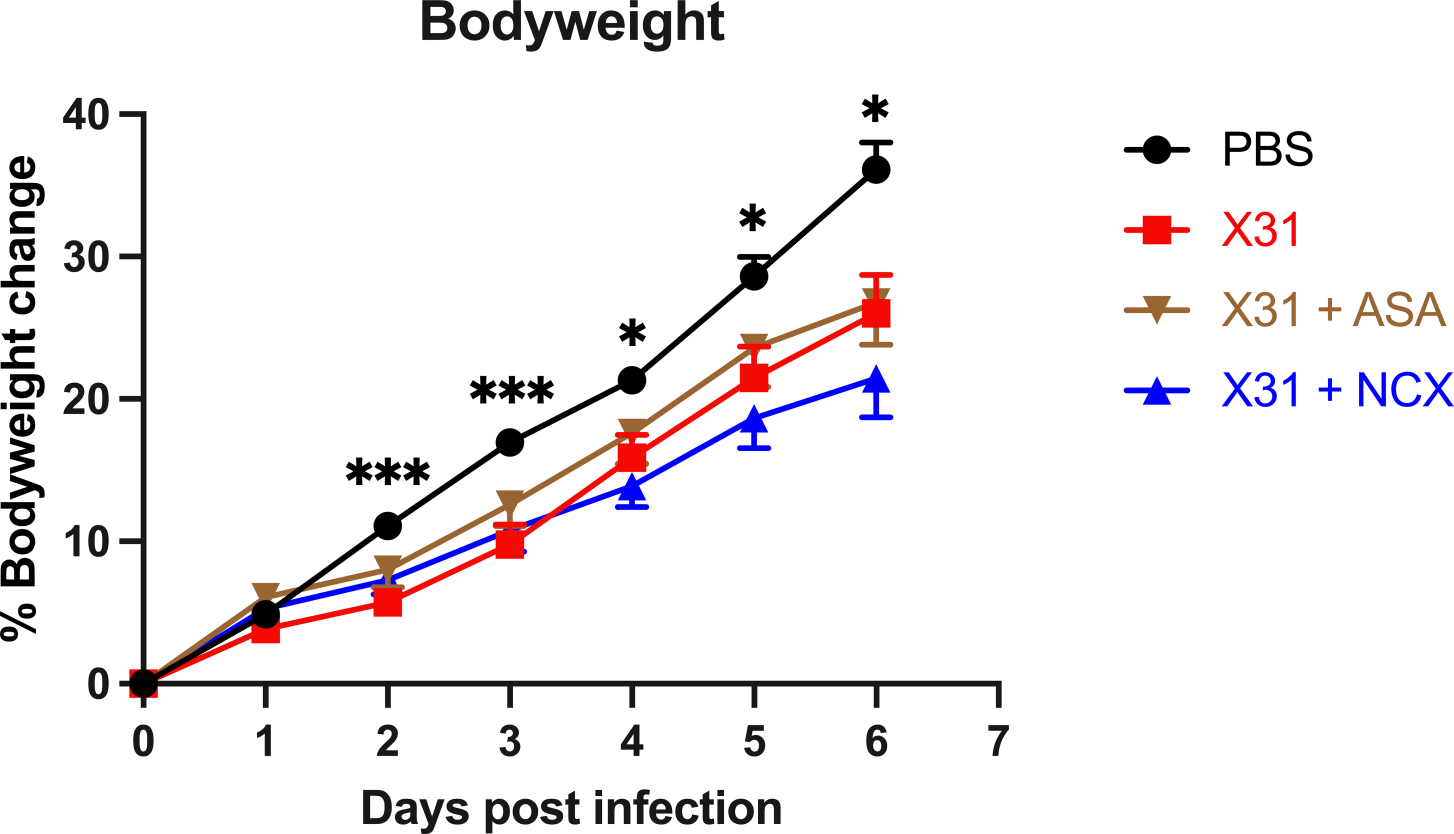
Analysis of dam weight gain following influenza A virus infection. Pregnant dams were infected at E12 with 10^4^ PFU of X31 virus or mock-infected with PBS. Dams were treated with 200µg/kg bodyweight of aspirin (ASA) or NCX4016 (NCX). Dams were weighed daily up until cull to understand how effectively the pregnancy was progressing. Data are represented by the mean ± SEM, with an n=19-24/group. Statistical analysis was performed using a two-way ANOVA, followed by a Dunnett’s multiple comparison’s test. *P<0.05, ***P<0.001 vs X31.

A key marker of the severity of X31 infection in pregnant dams is a reduced weight gain. X31 infected dams showed a significantly smaller weight gain throughout the course of infection up until day 6 post infection (d.p.i; E18) (**Figure 1**). The bodyweight gain of X31 infected dams, which were treated with ASA or NCX for up to 3 days post infection, was not significantly improved compared to X31 dams (**Figure 1**).

### Treatment with ASA or NCX4016 modified subsets of immune cell populations in the BALF in response to X31 infection, but had no effect on viral titres

**Figure 2:**
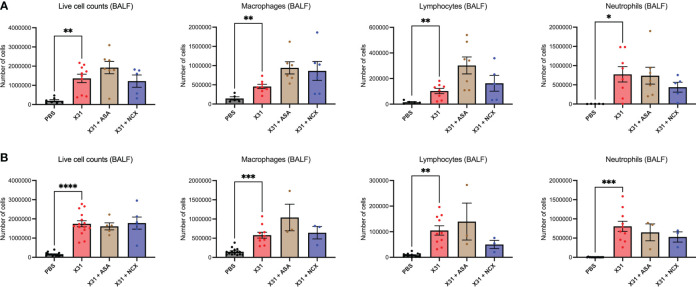
ASA and NCX4016 treatment modified IAV-induced BALF inflammation. Pregnant dams were infected at E12 with 10^4^ PFU of X31 virus and treated with either 200µg/kg bodyweight of aspirin (ASA) or NCX4016 (NCX). Dams were then culled at **(A)** 3 days or **(B)** 6 days post infection. BALF analysis including differential cell analysis of macrophages, lymphocytes, and neutrophils were conducted to investigate inflammation within the airways. Data are represented by the mean ± SEM, with an n=5-15/group. Statistical analysis was performed using a one-way ANOVA, followed by a Dunnett’s multiple comparison’s test. *P<0.05, **P<0.01, ***P<0.001, ****P<0.0001.

Pregnant mice were culled at 3- and 6 d.p.i representing the time points for peak lung and airway innate inflammatory response;peak viral burden (3 dpi); and the beginning of the adaptive immune response (6 dpi); in mice using this model system (8). Total live cell counts in the BALF were performed to measure infiltration of inflammatory cells into the airways at 3 and 6 dpi, and used as a marker for BALF inflammation. As expected, X31 infection significantly increased the number of total inflammatory cells in the BALF (**Figure 2**). Treatment with either ASA or NCX4016 had no significant effect on X31-induced BALF inflammation (**Figure 2**). In contrast, ASA and NCX4016 treatment influenced differential cell counts in the airways when compared to X31 dams. X31 infection significantly increased BALF macrophages, lymphocytes and neutrophils (**Figure 2**). ASA treatment of X31 infected dams did not affect BALF macrophages lymphocyte, and neutrophil numbers at both day 3 and day 6 post infection (**Figure 2**). There was also no effect by NCX4016 on macrophage, lymphocyte, and neutrophil infiltration at 3 and 6 dpi into the airways (**Figure 2**). It was noted that the effects of ASA and NCX-4016 on macrophage and lymphocytes counts differed in that ASA appeared to increase lymphocyte and macrophages in the lungs at 3 and 6 dpi while NCX did not. We suggest that this might be attributed to the NO-donation by NCX resulting in an anti-inflammatory effect, which is well established for NO (29). In the uninfected cohorts, ASA and NCX-4016 treatment had no effect on BALF inflammation, BALF macrophage, neutrophil and lymphocyte counts (**Supplementary Figure 1**).

**Figure 3:**
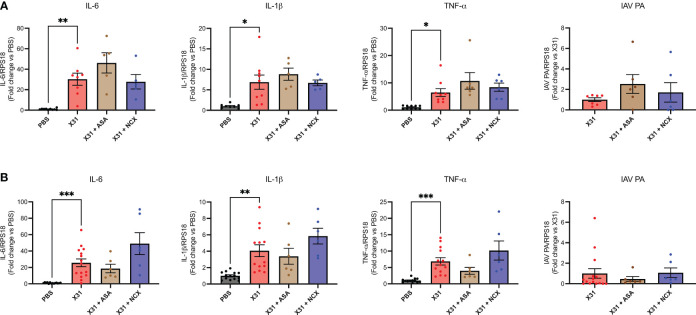
ASA and NCX4016 treatment had no effect on lung pro-inflammatory cytokine expression or viral titers following IAV infection. Pregnant dams were infected at E12 with 10^4^ PFU of X31 virus and treated daily with either 200µg/kg bodyweight of aspirin (ASA) or NCX4016 (NCX). Dams were then culled at **(A)** 3 days or **(B)** 6 days post infection. qPCR was performed to examine lung cytokine levels. Data are represented by the mean ± SEM, with an n=6-13/group. Responses are relative to RPS18 and expressed as a fold change above the control. Statistical analysis was performed using a one-way ANOVA, followed by a Dunnett’s multiple comparison’s test. *P<0.05, **P<0.01, ***P<0.001.

X31 infected pregnant mice showed significant increases in pro-inflammatory cytokines (IL-1β, IL-6 and TNF-α) within the lungs (**Figure 3**), as has previously been observed (8). Treatment with either ASA or NCX4016 had no effect the pro-inflammatory cytokine expression within the lungs (**Figure 3**). The IAV polymerase (PA) mRNA expression was examined in the lungs. Neither ASA nor NCX affected PA expression at 3 or 6 dpi (**Figure 3**).

### ASA or NCX4016 significantly improved endothelial dependent vascular function in the aorta of pregnant mice following X31 infection

**Figure 4:**
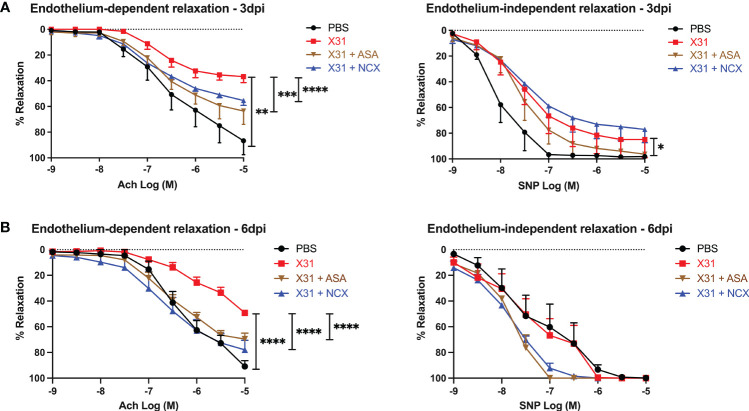
ASA and NCX4016 significantly reversed endothelial dysfunction at 3- and 6- days post infection. Pregnant dams were infected at E12 with 10^4^ PFU of X31 virus and treated daily with either 200µg/kg bodyweight of aspirin (ASA) or NCX4016 (NCX). Dams were then culled at either **(A)** 3 days or **(B)** 6 days post infection. Wire myography was used to determine endothelial dependent (Ach) and independent relaxation responses (SNP). Data are represented by the mean ± SEM, with an n=4-9/group. Statistical analysis was performed using a one-way ANOVA, followed by a Tukey’s multiple comparison’s test. *P<0.05, **P<0.01, ***P<0.001 and ****P<0.0001.

To investigate the effect of ASA and NCX4016 on vascular dysfunction caused by X31, we used wire myography to examine endothelial-dependent and -independent vascular function in isolated aortas. Aortas taken from X31 infected dams showed a significantly impaired response to Ach and SNP when compared to uninfected PBS dams at 3 dpi, which is consistent with previous observations (8). The response to Ach was only 35% when compared with the PBS group, which showed ~ 87% total relaxation to Ach (**Figure 4**; *P* = 0.0043). However, X31-infected pregnant dams that were treated with either ASA or NCX4016 showed a significant reversal of this impaired vascular phenotype. Maximal relaxation in response to Ach was 63% and 55% respectively for ASA and NCX4016 (*P* = 0.0003 and *P* < 0.0001 respectively). ASA treatment significantly improved the endothelial-independent relaxation to SNP at 3 dpi (**Figure 4**). NCX4016 treatment had no significant effect on the relaxation to SNP (**Figure 4**). As previously reported, X31 infection induces aortic endothelial-dependent dysfunction even up to 6 dpi in pregnant dams (8). At 6 dpi, a significant impairment in endothelial-dependent relaxation to Ach in aorta from X31 infected dams was observed (**Figure 4**). The response to Ach was ~49% compared to PBS dams which showed a 91% relaxation response to Ach (*P* < 0.0001). Treatment with either ASA or NCX4016 prevented this X31-induced dysfunction, with ASA and NCX4016-treated dams showing ~69 and ~78%, respectively in maximal relaxation (**Figure 4**). At 6 dpi, the SNP relaxation response was not impaired by X31 infection, consistent with our previous report (8). Both ASA and NCX4016 improved the relaxation response to SNP, although this did not reach statistical significance (**Figure 4**). Together, this data suggests that ASA and NCX4016 are effective at restoring X31 induced endothelial-dependent vascular dysfunction.

### Treatment with ASA and NCX4016 reduces viral dissemination and inflammation into the aorta of pregnant mice

**Figure 5:**
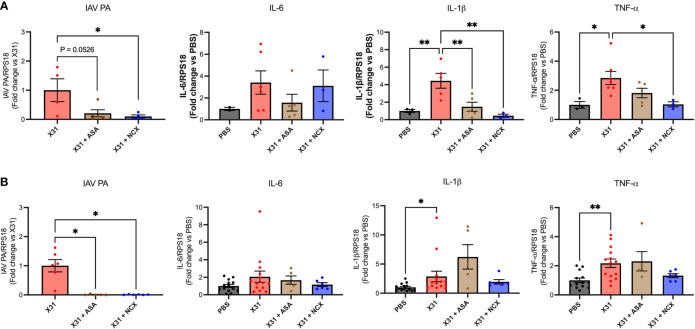
ASA and NCX4016 reduce viral titre and inflammation in the aorta after X31 infection. Pregnant dams were infected at E12 with 10^4^ PFU of X31 virus and treated daily with either 200µg/kg bodyweight of aspirin (ASA) or NCX4016 (NCX). Dams were then culled at **(A)** 3 days and **(B)** 6 days post infection. qPCR was conducted to investigate viral PA, IL-6, IL-1β and TNF-α expression within the aorta. Data are represented by the mean ± SEM, with an n=4-14/group. Responses are relative to RPS18 and expressed as a fold change above the control. Statistical analysis was performed using a one-way ANOVA, followed by a Dunnett’s multiple comparison’s test. *P<0.05, **P<0.01.

We have previously shown that X31 disseminates into the aorta along with the significant increase in inflammation characterised by the increased expression of pro-inflammatory cytokines (8). Therefore, to assess whether ASA or NCX4016 affected aortic inflammation and viral titres, we examined the expression levels of TNF-α, IL-6, IL-1β and IAV PA. Both ASA and NCX4016 treatment resulted in a decrease in the amount of virus present within the aorta when compared to the X31 alone group, suggesting that both treatments were able to reduce viral dissemination at 3 and 6 dpi (**Figure 5**). Analysis of Ct values of IAV PA mRNA transcripts in the aorta also showed similar reductions following ASA or NCX4016 treatment (**Supplementary Figure 3**). There was no significant change in IL-6 expression in the aorta with X31 infection and ASA and NCX4016 treatment at 3 and 6 dpi (**Figure 5**). In contrast, we also observed a significant reduction in pro-inflammatory cytokines TNF-α, and IL-1β within the vessel following ASA and NCX4016 treatment at 3 dpi (**Figure 5**). ASA and NCX4016 treatment also reduced TNF-α, and IL-1β at 6dpi, however this effect did not reach statistical significance. Taken together, our data suggests that ASA and NCX4016 reduces viral dissemination to the aorta and acutely suppresses the exacerbated vascular inflammation associated with the peak of viral tires and inflammation (3 dpi) during X31 infection in pregnant dams.

### ASA and NCX4016 improve offspring outcomes

**Figure 6:**
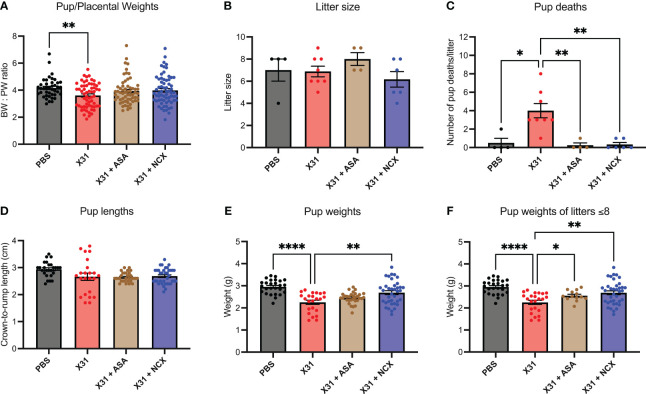
ASA and NCX4016 significantly improved pup/placental ratios, and pup survival rates post-delivery. Pregnant dams were infected at E12 with 10^4^ PFU of X31 virus and treated with either 200µg/kg bodyweight of aspirin (ASA) or NCX4016 (NCX). **(A)** Dams were then culled at E15 gestation to assess pup/placental ratios, n=42-72 pups/group. **(B–F)** In separate experiments, dams were allowed to deliver and litter sizes were recorded with pups then culled at postnatal day 5 (P5). Litters were monitored daily, and the number of dead pups were noted. At P5, pup crown-to-rump lengths and bodyweights were also taken. Data are represented by the mean ± SEM, with an n=23-37 pups/group Statistical analysis was performed using a one-way ANOVA, followed by a Dunnett’s multiple comparison’s test. *P<0.05, **P<0.01, ****P<0.0001.

Epidemiology and preclinical studies have shown X31 infection during pregnancy results in smaller birth weight offspring (30). Given that ASA and NCX4016 restored maternal vascular function that was impaired by X31 infection, we next sought to determine whether this reversal of vascular dysfunction by ASA and NCX4016 was associated with improved offspring outcomes. To interrogate this, we recorded pup to placental weight ratios at E15. In humans, the ratio of foetal bodyweight (BW) to placental weight (PW) is used as an indicator for placental insufficiency (31). A small BW : PW ratio could suggest impaired nutrient transfer from the placenta to support the foetus. In this study, we show that X31 infected dams had significantly smaller pup to placental ratios at E15 gestation, suggesting that maternal X31 infection results in an acute impairment of the placenta’s ability to effectively transfer nutrients to the pups. The pup to placental ratios in the ASA and NCX4016 treated groups were not significantly different when compared to PBS, indicating a prevention of placental dysfunction with ASA and NCX4016 (**Figure 6**). IAV-infected pregnant dams are associated with placental stress, as measured by the secretion of the antiangiogenic protein sFLT1 from the placenta (8). sFLT-1 into the maternal circulation promotes an exacerbated systemic inflammatory response in the maternal circulation. We also examined sFLT-1 levels with ELISA. IAV infection increased sFLT-1 in the plasma. This response was prevented by aspirin or NCX treatment (**Supplementary Figure 2**).

IAV infection of pregnant mice with 10^4^ PFU of X31, resulted in low rates of pup survival to postnatal day (P)5 age compared to PBS treated offspring (**Figure 6C**). To assess whether ASA and NCX4016 improved pup survival, we treated pregnant mice for 6 days (i.e. to E18) post X31 infection and then tracked the offspring. First, IAV infection did not influence the overall litter sizes. Litter sizes determined at day of delivery P0 were not affected by either ASA or NCX4016 treatment (**Figure 6B**). Of note, ASA treated mice had slightly larger litter sizes compared to the other groups however this was not statistically significant. We monitored dams and pups daily for 5 days post birth and then recorded pup weights and lengths at P5. X31 infected dams had poor offspring survival rates to P5, with dams frequently losing 100% of their litters (4/8 litters). When we compared this to ASA and NCX4016 treated dams, we observed significantly improved offspring survival rates. Furthermore, surviving P5 pups born from X31 infected dams weighed significantly less than pups from PBS dams (**Figure 6E**). When examining the effect of ASA and NCX4016 treated groups, both treatments showed significantly heavier pups than pups born from X31 infected dams, when unadjusted and adjusted for litter size effects by assessing bodyweights from litters with 8 or less pups (**Figure 6F**). When examining pup crown-to-rump length, we saw no significant differences between the groups (**Figure 6D**). Together, our offspring data suggests that initiating ASA or NCX4016 treatment immediately after X31 infection can successfully improve birth outcomes and survival rates in the offspring.

## Discussion

This study is the first to provide evidence that low dose ASA and NCX4016 treatment are effective in reversing maternal vascular dysfunction and improving offspring outcomes following IAV infection during pregnancy in mice. The study also provides evidence that the suppression of the vascular dysfunction by low dose ASA and NCX4106 is likely to involve suppression of viral dissemination of IAV to the aorta and the consequent inflammation.

The endothelium is critical in maintaining a proper homeostatic balance, that involves the regulation of vascular tone and permeability, coagulation and inflammatory responses (32). The pathophysiology of multiple cardiovascular diseases including atherosclerosis and coronary artery disease involves inflammation and dysfunction of the endothelium (33). Endothelial dysfunction is also a hallmark of preeclampsia, a hypertensive disease in pregnancy (34). Of relevance to this study, endothelial dysfunction was also observed with IAV infection during pregnancy (8). In preeclampsia, maternal endothelial dysfunction is associated with impaired blood flow to the placenta resulting in poor foetal outcomes (35). In this study, we showed that ASA and NCX4016 treatment prevented vascular dysfunction associated with maternal IAV infection. We hypothesise that the improvement in endothelial function in the aorta, will improve placental blood flow and restore its function. Our present study did not address blood flow directly, as this warrants a separate comprehensive analysis of placenta blood flow, aortic stiffening and endothelial function of placental microvessels. Furthermore, ASA has been shown to cross the placental barrier (36) and so it is feasible that it may have had an effect on placental angiogenesis, particularly with the observed reversal of intrauterine growth restriction. We examined serum sFlt-1 in our model and observed significant reductions in sFlt-1 with drug treatment (**Supplementary Figure 2**), which may be a surrogate marker for promoting angiogenesis (37). Future studies should examine whether gestational influenza and aspirin and NCX4016 influence neo-angiogenesis during pregnancy.

ASA presents as an attractive therapeutic that is already reported to be safe in pregnancy (38). However, currently, there is a lack of evidence base for the utility of ASA during IAV infection in pregnancy. Some studies have suggested that ASA has anti-viral activities against H1N1 infection *in vitro* (25, 39). However, the concentrations of ASA used *in vitro* that possessed anti-viral activity were very high (EC50 of ~ 660μM). Whilst these concentrations might be achieved *in vivo* with anti-inflammatory doses of ASA (i.e. ~1.2 g/4-6hr), these doses are far higher than those used in the present study, which were 200 μg/kg/day (~1.4 mg for a 70kg person) similar to what is prescribed to pregnant women. This dose of ASA is very low and therefore we predict that the vascular inflammatory effects of ASA in this study are not due to an anti-viral effect. We propose that the most likely mechanism of action of low dose ASA in the current study, is suppression of COX, particularly the COX1 isoform. COX1 plays an important role in regulating prostaglandin production which regulates platelet activation. ASA treatment irreversibly blocks platelet COX1 affecting both clotting pathways and vasodilatory and vasoconstrictor pathways (40). We hypothesize that ASA suppresses platelet-dependent inflammatory and vascular effects at the endothelium during an IAV infection. In this regard, we have previously shown that IAV infection during pregnancy triggers increased accumulation of monocytes, neutrophils and T cells to the aorta, in addition to increased pro-inflammatory cytokine expression and oxidative stress formation at the vessel wall and surrounding perivascular fat (8). This increase in vascular inflammation and oxidative stress during IAV infection is likely to compromise endothelial function and might even result in damaged endothelium. Previous studies have shown that IAV infection can induce platelet interactions with the endothelium (41), which can lead to platelet aggregation, NETosis and vascular inflammation. Indeed, inhibiting this platelet-endothelium interaction may therefore prevent endothelial dysfunction. Following endothelial cell damage, exposure of platelets to the sub-endothelial space and collagen fibres will result in their attachment to the vessel wall and their subsequent activation. This activation triggers the expression of glycoprotein IIb/IIIa (ITGA2B) by the platelets which bind to von Willebrand Factor (vWF) or fibrin (42) and trigger the aggregation of platelets at the site of injury. We suggest that by irreversible suppression of platelet COX1, ASA inhibits platelet aggregation in the vessel wall caused by IAV infection via suppression of thromboxane production (43).

Although we did not directly measure aortic endothelial cell injury in this model, we observed significant reductions in the capacity of the aortic vascular endothelium to stimulate vascular relaxation, as well as increases in pro-inflammatory cytokines, strongly suggesting vascular endothelial cell injury. Other *in vitro* studies have shown influenza can infect the pulmonary vasculature, which can contribute to increased permeability and vascular leak (44). Future studies should assess whether gestational influenza infection does cause aortic endothelial cell injury.

In addition to COX inhibition, ASA has been shown to promote vasorelaxation via stimulation of endothelial nitric oxide (NO) release through eNOS acetylation, a mechanism that is independent of COX (24). Endogenous NO is critical for vascular function, supressing cellular inflammation, inhibiting platelet aggregation, and regulating signalling pathways (45, 46). An action of ASA on eNOS may explain the anti-inflammatory effects at the aorta and the improvement in endothelial function. NCX4016 is a NO releasing ASA moiety, which is likely to possess COX inhibitory and additionally, NO-mediated effects. These NO-mediated effects include inhibition of inflammatory cytokine synthesis (47), inhibition of leukocyte adherence to the vascular endothelium (48) and the direct promotion of vasodilation. The finding that NCX4016 did not further promote endothelial cell relaxation was indeed surprising, as we expected enhanced vascular relaxation. There are limited studies on the metabolism of NCX4016 in mice, however those that exist suggest that NO and acetylsalicylic acid (the active component in aspirin) exert their effects simultaneously in mouse models, and so it possible that the addition of excess NO to the vasculature did not further protect from viral inflammation (49).

Preeclampsia is one of the most well studied cardiovascular diseases during pregnancy. It is the primary cause of death in one in four neonates with effects such as increased risk of stillbirth, intrauterine growth restriction, premature birth, and poor neurodevelopmental outcomes (50). Low-dose ASA of between 50 and 150 mg/day is commonly prescribed to high-risk pregnant women to prevent the development of preeclampsia (51). We have identified in our study that not only was ASA effective at reversing vascular dysfunction, but that it also significantly improved foetal outcomes, providing further evidence of the impact of IAV-induced maternal vascular dysfunction on healthy foetal development. We observed greater foetal bodyweight to placental weight ratios *in utero* during the IAV infection, signifying an improvement in placental sufficiency during the pregnancy. There was also a suppression of placental derived sFLT-1 by aspirin and NCX-4016 treatment signifying a reduction in placental stress. This improved *in utero* health trajectory was evident in the offspring outcome analysis which demonstrated marked improvements in 5 day old offspring weights born from ASA and NCX4016 infected dams. This is important as low birth weights for gestational age is a well-known risk factor for the development of diseases later in life such as cardiovascular complications and diabetes (52). Interestingly, the dose of ASA used in this study (200 µg/kg bodyweight) is comparable to that prescribed to pregnant women for pre-eclampsia.

Although ASA and NCX4016 treatment prevented IAV dependent vascular effects and restored offspring outcomes, the analysis of the effect of drug treatment on the severity of the lung infection and inflammation suggests the local inflammatory response was largely preserved. Indeed, this is exemplified in the viral titre levels and airway inflammation being unaffected by ASA and NCX4016 treatment, suggesting a lack of anti-viral effect of these treatments in the airways. It is possible that the bioavailability of ASA and NCX4016, given such as small dose was used, was insufficient to reach the lungs and have an effect there, when compared to the aorta. Perhaps other methods of treatment may be investigated, such as inhaled or intranasal to ensure that ASA and NCX reach the site of infection. These findings also suggest that the suppression of viral dissemination to the aorta by ASA and NCX4106 is not due to a local lung viral clearance mechanism but to suppression of mechanisms of viral dissemination. Future studies are needed to decipher how IAV is being disseminated from the lungs and how ASA treatment prevents this from occurring. Therefore, it is possible that the effects of ASA and NCX4106 are dual, i.e. on viral dissemination per se and on the vascular inflammatory effects caused by the virus after it has disseminated to the peripheral arteries.

In addition to viral infections, both bacterial and parasitic infections during pregnancy place the mother and developing foetus at significant risk of adverse outcomes, such as miscarriage and intrauterine growth restriction (53, 54). Parasitic and bacterial pathogens can also cause placental inflammation which may cause alterations in foetal and maternal haemodynamics. It remains to be determined whether low dose aspirin can have similar therapeutic effects during these infections and if it similarly represents as an attractive, cost effective and safe therapeutic for these maternal infections as well.

This study provides valuable insight into the administration and effects of ASA treatment for IAV infection during pregnancy. Despite vaccination being the best means of protection, vaccination rates remain low in the pregnant population, and issues with administering anti-viral medication at the correct treatment window are challenging. We have shown that low dose ASA can prevent IAV-induced vascular dysfunction in pregnant dams, reduce systemic viral dissemination and improve offspring survival. Given that ASA treatment is safe during pregnancy and is currently used in clinics to treat preeclampsia in high-risk pregnant women, translational studies to assess the efficacy of using low dose ASA in treating pregnant women with IAV infection should be considered and could be easily implemented in the clinic.

## Data availability statement

The original contributions presented in the study are included in the article/**Supplementary Material**. Further inquiries can be directed to the corresponding authors.

## Ethics statement

All animal experimentation was approved by the Animal Ethics Committee (AEC) at RMIT University (Animal Ethics Committee Number 1801) and as specified by the guidelines of the Australian Code of Practice for the Care of Experimental Animals and National Health and Medical Research Council of Australia (NHMRC).

## Author contributions

MC-S: Writing – review & editing, Writing – original draft, Methodology, Investigation, Formal analysis, Conceptualization. SL: Writing – review & editing, Writing – original draft, Methodology, Investigation, Funding acquisition, Formal analysis, Conceptualization. OO: Writing – review & editing, Writing – original draft, Investigation. JE: Writing – review & editing, Writing – original draft, Investigation. MM: Writing – review & editing, Writing – original draft, Investigation. FL: Writing – review & editing, Writing – original draft, Investigation. KB: Writing – review & editing, Writing – original draft, Investigation. SB: Writing – review & editing, Writing – original draft, Conceptualization. RV: Writing – review & editing, Writing – original draft, Conceptualization. RB: Writing – review & editing, Writing – original draft, Conceptualization. DB: Writing – review & editing, Writing – original draft, Funding acquisition. JO’L: Writing – review & editing, Writing – original draft, Funding acquisition, Conceptualization. SS: Writing – review & editing, Writing – original draft, Supervision, Resources, Project administration, Methodology, Investigation, Funding acquisition, Formal analysis, Data curation, Conceptualization.
