# Supplementary material for: Low dose aspirin prevents endothelial dysfunction in the aorta and foetal loss in pregnant mice infected with influenza A virus
Source: Front Immunol. 2024 Apr 4;15:1378610. doi: 10.3389/fimmu.2024.1378610 (PMC11024306; doi:10.3389/fimmu.2024.1378610)
Supplement: Supplementary file 1 [file DataSheet_1.docx]

**Supplementary Figure 1: ASA and NCX4016 treatment did not modify immune cell recruitment in BALF**

Pregnant dams were mock-infected with PBS and treated with either 200µg/kg bodyweight of aspirin (ASA) or NCX4016 (NCX). Dams were then culled at 3 days post infection. BALF analysis including differential cell analysis were conducted to investigate inflammation within the airways. Data are represented by the mean ± SEM, with an n=5-6/group. Statistical analysis was performed using a one-way ANOVA, followed by a Tukey’s multiple comparison’s test.

**Supplementary Figure 2: ASA and NCX4016 treatment suppressed IAV-induced circulating sFLT-1 levels**

Pregnant dams were infected at E12 with 10^4^ PFU of X31 virus and treated with either 200µg/kg bodyweight of aspirin (ASA) or NCX4016 (NCX). Dams were then culled at 6 days post infection. Circulating levels of sFLT-1 was quantified by ELISA. Data are represented by the mean ± SEM, with an n=6-13/group. Statistical analysis was performed using a one-way ANOVA, followed by a Fisher’s LSD multiple comparison’s test. **P*<0.05, ***P*<0.01.

**Supplementary Figure 3: Ct values of IAV PA transcripts in the aorta of infected mice.**

Pregnant dams were infected at E12 with 10^4^ PFU of X31 virus and treated with either 200µg/kg bodyweight of aspirin (ASA) or NCX4016 (NCX). Dams were then culled at 3 or 6 days post infection (DPI). Viral titres were measured by qPCR using primers specific against the IAV PA gene. Cycle threshold (Ct) values for IAV PA in the aorta is presented as the mean ± SEM, with an n=4-6/group. Statistical analysis was performed using a one-way ANOVA, followed by a Tukey’s multiple comparison’s test. *****P*<0.0001.
